# Supplementary material for: Telehealth equity and access communication skills pilot simulation for practicing clinicians
Source: PLoS One. 2025 Jan 6;20(1):e0302804. doi: 10.1371/journal.pone.0302804 (PMC11703036; doi:10.1371/journal.pone.0302804)
Supplement: S1 Appendix — (DOCX) [file pone.0302804.s001.docx]

After experiencing this curriculum, participants should be able to:

Adjust physical characteristics (physical space, camera, lighting, microphone) *and* use words/language/dialogue to ensure that the patient conveys that they are experiencing a safe environment for a video-based telehealth encounter.

Create a therapeutic rapport via telehealth by using verbal communication that explains and clarifies activities that are part of the telehealth encounter (provider actions/examination techniques) *and* non-verbal behaviors that express listening/empathy and attend to any transmission delays.

Apply language/word usage that partners with the patient to ensure that any risks or unsafe conditions related to the patient’s care are mitigated and mutually understood by the provider and the patient

Inquire about, and include as appropriate, a patient’s family/social supports to enhance care during and after the telehealth encounter.

Use clarifying and confirming language to ensure mutually understood post-encounter care plans, and the accessibility of care needs as appropriate, before concluding a telehealth encounter.
