# Supplementary material for: Telehealth equity and access communication skills pilot simulation for practicing clinicians
Source: PLoS One. 2025 Jan 6;20(1):e0302804. doi: 10.1371/journal.pone.0302804 (PMC11703036; doi:10.1371/journal.pone.0302804)
Supplement: S2 Appendix — (DOCX) [file pone.0302804.s002.docx]

**Case A:**

**Blood pressure question**

**STANDARDIZED PATIENT SCRIPT:**

Chief concern: “I think I need my blood pressure medicines adjusted.”

**HPI:**

You are Jaime Hatch, a 56-year-old [female/male] with a history of hypertension. You are calling the Telehealth Line for an urgent visit because you think you may need your blood pressure medicines adjusted. You have been taking the same prescription for the last 15 years, but recently you have been having annoying headaches and you think your blood pressure must be up.

You started to develop dull, throbbing headache “all over your head” last week. You have had 3 or 4 of these headaches in the last week. When present, it is 2/10 in severity. You notice that the headache seems to come on gradually at the end of the workday after you have arrived home from work as a CNA. It seems to get worse through the evening while you are making dinner, cleaning up the kitchen and trying to get your 2 grandchildren (ages 3 and 18 months) bathed and ready for bed. By the time the kids are asleep, it’s almost 9:30 pm and the headache is most intense, to the point that you often must take 2 Tylenol and 3 Motrin to get the headache to decrease in intensity. You are taking both medications at the same time, in line with dosing recommendations on the box. Sometimes you just “give up” and go to bed, hoping that the next day will be better. You know that high blood pressure can cause headaches, so you have been checking your blood pressure frequently at work and wonder if you need a medication adjustment.

Your blood pressure measurement ranges: 120s/85 up to 150s/90

You have 2 main concerns:

1. Your blood pressure medications are no longer working. You think they need to be changed.
2. Your headaches are making it hard for you to manage your work, grandchildren, and online school: “What if I have a stroke? There’s no one to take care of my grandkids.”

You have 1 main question:

“Can you please have new blood pressure medicines?”

*If asked about discomfort or other symptoms:*

You do not have change in vision, difficulty speaking, numbness or weakness in your face or extremities.

You do not have nausea or dizziness.

You do not have any illness, fever, sore throat, cough, difficulty breathing, or swelling in your legs

You have not had any head injury or other trauma.

**Past Medical History:**

Hypertension

Arthritis in the knees

**Medications:**

HCTZ 25 mg daily

Valsartan 160 mg daily

**Over-the-Counter Medications:**

Motrin for knee pain, 1 – 2 pills daily

Tylenol and Motrin for headaches

**Allergies:**

None

**Social History:**

You work as a CNA at a local nursing home. You live in an apartment with your grandchildren. Their mother, your daughter, died 6 months ago in a car accident and their father has been “out of the picture” for over a year. The children stay with a neighbour during the day while you are at work.

Two weeks ago, the nursing home announced that they will be cutting staff in the next few months during planned construction of the facility. You are worried about losing your job and being able to pay rent and buy food and clothes for your grandkids.

Tobacco use: You do not smoke

Alcohol use: You do not use alcohol

Illicit substance use: no other drug use

*If asked about other supports:*

“I can’t bother my neighbour any more than I already do.”

**Exam:** You appear worried, slightly depressed affect, but in no distress. You are able to speak in full sentences and the neurologic exam is normal (any and all maneuvers are performed without difficulty).

**Vital Signs:**

You checked your blood pressure and heart rate at the pharmacy across the street 1 hour ago. The pharmacist had a portable pulse oximeter that he allowed you to use.

Blood pressure: 148/85

Pulse: 82

Respiratory Rate:

Oxygen saturation: 97%
