# Supplementary material for: Telehealth equity and access communication skills pilot simulation for practicing clinicians
Source: PLoS One. 2025 Jan 6;20(1):e0302804. doi: 10.1371/journal.pone.0302804 (PMC11703036; doi:10.1371/journal.pone.0302804)
Supplement: S3 Appendix — (DOCX) [file pone.0302804.s003.docx]

**Case B:**

**Asthma question**

**STANDARDIZED PATIENT SCRIPT:**

Chief concern: “I think I need my asthma medicines adjusted.”

**HPI:**

You are Ryan Poirier, a 56-year-old [female/male] with a history of asthma. You are calling the Telehealth Line for an unscheduled visit because you wonder if you may need your asthma medicines adjusted. You have been only previously needed an albuterol MDI infrequently, roughly once a year, in the last 15 years, but recently you had to use your inhaler for three full days (which is new) and you think your asthma might need more care than it used to.

You started to develop a tightness in your chest and associated shortness of breath “just the same as my asthma always is” last week. This lasted 2-3 days in total and now is fully resolved. Importantly, you have NO shortness of breath today and you feel your breathing is 100% normal today. Your symptoms seem to reliably come on in the winter/cold weather, when around lawns being mowed, or if you’re around dogs for too long. You had been diagnosed with asthma a long time ago as a high schooler, you’re not sure if you did any pulmonary function tests. Your symptoms used to be very well controlled just with albuterol as needed and would turn around within 1-2 days, but the last episode lasted much longer and caused you to miss work, which can’t happen again.

If asked, you did not have any fevers, you did have a slight cough but mostly noticed wheezing and a tightness feeling. No productive cough. No chest pain. You tested negative for COVID on your at-home tests. Importantly, you feel very well now, but you never want to see this happen again.

Your asthma has never required admission to the hospital, never required intubation. You have never seen a pulmonologist. You don’t know if you’ve ever needed a steroid burst before. If asked, you’re not sure what a nebulizer is.

Your blood pressure measurement was: 131/85, and your last pulse oximeter check (if asked) was 97% today at the pharmacy.

You have 1 main concern:

Albuterol alone might not be enough any longer. Do I need to have some new medications so that I don’t miss work anymore?

You have 1 main question:

“What additional life changes should you make to control your asthma?”

Additional ROS:

You do not have difficulty speaking, numbness or weakness in your face or extremities. You are currently asymptomatic.

You do not have any illness, fever, sore throat, cough, difficulty breathing, or swelling in your legs.

You have not had any head injury or other trauma.

**Past Medical History:**

Asthma

**Medications:**

albuterol MDI every 4 hours as needed for wheezing

**Over-the-Counter Medications:**

None

**Allergies:**

None

**Social History:**

You work in construction and have recently been working nights repaving a high-traffic road. You are on this telehealth call after a night shift. Your last asthma exacerbation caused friction at work, and your boss is not very accommodating—you had to call in because you were not feeling up to doing the physical labor required at work. You live 30 miles outside of Boston and your drive can take up to 1.5 hours, depending on traffic in the evening. You are concerned about the cost of an additional daily asthma controller medication. You live alone, and your family lives outside of the state.

Tobacco use: Former one pack per day smoker, but quit 5 years ago

Alcohol use: You do not use alcohol

Illicit substance use: no other drug use

**Exam:** You appear in no distress. You are able to speak in full sentences. You are not in any distress at all right now and you can easily comply with any exam maneuvers requested of you.

**Vital Signs:**

You checked your blood pressure and heart rate at a local pharmacy yesterday. The pharmacist had a portable pulse oximeter that he allowed you to use.

Blood pressure: 131/85

Pulse: 82

Respiratory Rate: 14

Oxygen saturation: 97%
