# Supplementary material for: Telehealth equity and access communication skills pilot simulation for practicing clinicians
Source: PLoS One. 2025 Jan 6;20(1):e0302804. doi: 10.1371/journal.pone.0302804 (PMC11703036; doi:10.1371/journal.pone.0302804)
Supplement: S5 Appendix — (DOCX) [file pone.0302804.s005.docx]

How well does the clinician:

|  | | | |
| --- | --- | --- | --- |
| **A. Set up the environment (includes the following):** | **Did not Perform** | **Performed** | **N/A** |
| 1. Ensures my privacy by making sure that my space is private for me |  |  |  |
| 1. Ensures my privacy by making sure and indicating they are in a private space for their conversation with me (e.g., nobody else can hear our conversation on their end) |  |  |  |
| 1. Avoids background noise |  |  |  |
| 1. Uses appropriate lighting so that I can see them |  |  |  |
| 1. Turns off other applications (e.g., no other notification noises from emails or messages) |  |  |  |
| 1. Adjusts camera to eye level so that I can see their face |  |  |  |
| 1. Dresses professionally |  |  |  |
| 1. Begins information exchange by creating relaxed, empathetic environment that promotes good exchange between myself (the patient) and clinician |  |  |  |
| *Additional Comments:* | | | |
|  | | | |
| **B. Communication, Access, and Equity (includes the following):** | **Did not Perform** | **Performed** | **N/A** |
| 1. Uses non-judgmental statements when communicating with me |  |  |  |
| 1. Narrates and explains their actions (e.g., if they need to look at another screen while on the visit) |  |  |  |
| 1. Speaks clearly and deliberately so that I can understand |  |  |  |
| 1. Uses non-verbal language to show they are listening to me |  |  |  |
| 1. Uses pauses to facilitate bilateral communication (listening to me, observing me), allowing patient to contribute to information exchange |  |  |  |
| 1. Suggests escalation of care (e.g., go to the ED, visit in-person) if clinician believes I am unsafe (or I express that I feel unsafe) with distance care plan or in my current environment |  |  |  |
| 1. Ensures that my care is concordant with my preferences and values |  |  |  |
| 1. Explores whether I have social supports and incorporates them as able (if in line with my wishes) |  |  |  |
| 1. Thoroughly and accurately educates me about my illness, its management, and possible consequences with sensitivity to my concerns and preferences |  |  |  |
| 1. Verbalizes and clarifies post-encounter plans for my care |  |  |  |
| 1. Ensures that I have access to resources that will support my post-encounter care |  |  |  |
| *Additional Comments:* | | | |
|  | | | |
| **C. Gather Information (includes the following):** | **Did not Perform** | **Performed** | **N/A** |
| 1. Adjusts physical examination to the virtual environment |  |  |  |
| 1. Guides the patient through physical exam maneuvers |  |  |  |
| 1. Collects/uses the data captured by the patient (e.g., vital signs such as heart rate or where the patient reports pain) |  |  |  |
| *Additional Comments:* | | | |
