# Supplementary material for: Telehealth equity and access communication skills pilot simulation for practicing clinicians
Source: PLoS One. 2025 Jan 6;20(1):e0302804. doi: 10.1371/journal.pone.0302804 (PMC11703036; doi:10.1371/journal.pone.0302804)
Supplement: S6 Appendix — (DOCX) [file pone.0302804.s006.docx]

**Post-Session Survey**

**Simulated Telehealth Experience**

**1. Please indicate your agreement with the following statements.**

|  | Strongly Disagree | Somewhat Disagree | Neutral | Somewhat Agree | Strongly Agree |
| --- | --- | --- | --- | --- | --- |
| I can describe at least two ways to adjust physical characteristics (physical space, camera, lighting, microphone) to ensure that the patient experiences a safe environment for a video-based telehealth encounter. |  |  |  |  |  |
| I can describe at least two ways that I can use words/language/dialogue to ensure that the patient experiences a safe environment for a video-based telehealth encounter. |  |  |  |  |  |
| I can give two examples of techniques to create a therapeutic rapport via telehealth by using verbal communication techniques and nonverbal behaviors. |  |  |  |  |  |
| I feel confident applying language that partners with the patient to ascertain and mitigate any risks or unsafe conditions related to the patient’s care. |  |  |  |  |  |
| I can describe at least two ways to inquire about and include a patient’s family/social support to enhance care during and after a telehealth encounter. |  |  |  |  |  |
| I can describe language techniques to ensure mutually understood post-encounter care plans with my patient and the accessibility of care needs *before* concluding a telehealth encounter. |  |  |  |  |  |

**2. How likely are you to recommend this Simulated Telehealth Experience to your colleagues?**

**Not at all likely Extremely likely**

**0 1 2 3 4 5 6 7 8 9 10**

**3. Please provide any further feedback about the experience.**
