# Supplementary material for: Telehealth equity and access communication skills pilot simulation for practicing clinicians
Source: PLoS One. 2025 Jan 6;20(1):e0302804. doi: 10.1371/journal.pone.0302804 (PMC11703036; doi:10.1371/journal.pone.0302804)
Supplement: Attachment — Submitted filename: Feedback Reviewer_20240527.docx [file pone.0302804.s008.docx]

Feedback from Reviewer:

Thank you very much for the opportunity to peer-review your article “Telehealth Equity and Access Communication Skills Pilot Simulation for Practicing Clinicians”. It is very well written and aim of this study quite relevant to the field. .

However, I do have some concerns regarding the reporting of the intervention being tested and display of the results and recommend therefore major revision regarding the following comments, divided into the sections of the article.

I look forward to your revision. Thank you very much.

**Style:**

There are some minor sentences/words where I suggest different grammar use or sentence style:

- Page 4, lll 63-65: “Our team’s previous experience with video-based simulated 64 telehealth encounters demonstrated that practicing clinicians believe that this type of program 65 builds confidence and skills in the use of the telehealth modality [22].” Suggestion: “Our team’s previous experience with video-based simulated telehealth encounters indicated that practicing clinicians perceive these type of program as enhancing their confidence and skulls in using telehealth modality [22].”
- Page 4, ll 65-69: “We focused on 66 communication skills for this study because we recognized that there is significant risk of perpetuating inequity in healthcare, and telehealth may add an additional layer of complexity for these interactions that prior formal communications training may not have adequately addressed.” Suggestion: “We focused on communication skills development in this study as we recognized the significant risk of perpetuating inequity in healthcare, and telehealth may add an additional layer of complexity to these interactions that prior formal communications trainings may not have adequately addressed [30–34].
- Page 6, l 115: “intentionally crafted by our …”, Suggestions: “intentionally developed …”
- Page 6, line 118: “… and participating in online school.”. Who was participating in an online school? The grandmother? For me as a non US speaker, this would be bit unusual as “grandmothers do not go to school anymore. Maybe using “participating in an e-learning college programme.” If the grandkids participate in the online school, I would describe it as following. “ … caring for their grandchildren, who participate in an online school programme.”

**Methods:**

- General thought: did you pilot test the cases prior to the study? If not, please state. Also, were patients involved in the development?
- How many SPs did you plan on training and to be part of the study? Please describe how you selected these and which criteria were applied
- Instrument Design: How did you develop the SP survey? Can you please provide us with a bit more information here, e.g. how many items, what is the foundation of the development? Also, did you test the instruments before using it in this study?
- Curriculum development and teaching: I am missing the section of how the curriculum looked like and how it was thought? This in my opinion is the most important part of this pilot study, as it is the new intervention being developed and this is completely missing besides Appendix A with the objectives. Please provide an additional section on this in your methods section. Thank you.

**Results:**

- The way you present the results in table 2 with the SE values as well as p-values is a bit unusual and for me confusing. Could you please not provide results in the table in a more standardized statistically way, e.g. not repeating SE within the cells/results, having an additional p-value column or marking it by * or ** and giving annotations below the table?
- I also have trouble understanding what the wording “Percentage who performed” represents. Does this mean, 26,7% showed the skill “Ensures my privacy by making sure that my space is private for me”? And then the way the p-value is tucked under case 2. Please update the table in a more statistically sound way, e.g.,, with the question if the n differed between pre- and post-test? If not, than you can delete the post-test column and info behind n
- Also, I miss for the t-test the t-value, df…could you please report these?

Table 2

| Skill/Item | Pre-test (%, [SD]) | Post-test (%, [SD]) | n (Pre-test) | N (Post-test) | t-value (df) | p-value |
| --- | --- | --- | --- | --- | --- | --- |
| Skill 1 | 26.7 [68.2] |  | 30 | 28 | xxx | < 0.05 |

Annotation: SD – Standard Deviation, df – degrees of freedom

- I do have the same feedback for table 3, please adjust especially the last item of the recommendation into a fourth table, as this way it is really difficult to read and understand.

**Discussion:**

- Will give peer review once method and results section is clear to me as I do have informations about the intervention yet. Thank you.
