# Supplementary material for: Telehealth equity and access communication skills pilot simulation for practicing clinicians
Source: PLoS One. 2025 Jan 6;20(1):e0302804. doi: 10.1371/journal.pone.0302804 (PMC11703036; doi:10.1371/journal.pone.0302804)
Supplement: Attachment — Submitted filename: Response to Reviewers.docx [file pone.0302804.s009.docx]

Dear Editor,

Thank you for your careful assessment of our manuscript, titled "Telehealth Equity and Access Communication Skills Pilot Simulation for Practicing Clinicians." We appreciate the constructive feedback and the opportunity to revise our work. We have carefully addressed each of your comments, as well as those provided by Reviewer #1, and believe that the manuscript has significantly improved as a result.

Below, we provide a point-by-point response to your comments, including additional editorial requirements, and to those from Reviewer #1, as well as responses to additional questions posed by Reviewer #1:

**1. Manuscript Language and Clarity**
*Editor Comment*: The manuscript needs thorough language refinement for clarity, coherence, and readability. Consider professional editing services.

*Response*: We believe that the recommendations provided by Reviewer #1 have substantially enhanced the clarity and coherence of the manuscript. The revisions made in response to these comments have addressed areas requiring improvement, and we have reviewed the entire manuscript with careful attention to readability. Given that all authors are primarily English-speaking, we are confident that the manuscript meets appropriate standards of language and clarity. Should the editorial team identify specific areas that require further refinement, we would be grateful for additional guidance.

**2. Recent References**
*Editor Comment*: Add more recent references to better support the research gap. Ensure relevant studies, especially from the last five years, are included.

*Response*: We have conducted a thorough review of the literature and made updates to include the most recent and relevant studies available. In some instances, we have noted that the data referenced represents the latest information currently accessible. Additionally, we have added numerous recent references that have been published since the initial submission of the manuscript. If there are specific key references that the reviewers or editors believe should be included, we would welcome their guidance in further improving the citation base.

**3. Data Presentation and Results Interpretation**
*Editor Comment*: Improve data presentation with clearer visuals like tables or figures. Enhance the interpretation by aligning results with the research objectives and ensure the discussion is distinct from the results section.

*Response*: We have made significant improvements to the data presentation, specifically by revising the tables to enhance clarity and ensure consistency in the statistical information. The thoughtful comments provided by Reviewer #1 have been instrumental in guiding these revisions. We believe that the updated tables and the refined data presentation align more effectively with our research objectives. We believe that the discussion and results sections were already distinct; however, we are open to making further adjustments if needed upon revision.

**Reviewer #1 Comments and Responses**

**Style**:

- *Comment*: There are some minor sentences/words where I suggest different grammar use or sentence style:
  - Page 4, ll 63-65: "Our team’s previous experience with video-based simulated telehealth encounters demonstrated that practicing clinicians believe that this type of program builds confidence and skills in the use of the telehealth modality [22]." Suggestion: "Our team’s previous experience with video-based simulated telehealth encounters indicated that practicing clinicians perceive these types of programs as enhancing their confidence and skills in using telehealth modality [22]."
  - *Response*: We have updated the sentence to reflect the suggested changes, improving clarity and readability as recommended.
  - Page 4, ll 65-69: "We focused on communication skills for this study because we recognized that there is significant risk of perpetuating inequity in healthcare, and telehealth may add an additional layer of complexity for these interactions that prior formal communications training may not have adequately addressed." Suggestion: "We focused on communication skills development in this study as we recognized the significant risk of perpetuating inequity in healthcare, and telehealth may add an additional layer of complexity to these interactions that prior formal communications training may not have adequately addressed [30–34]."
  - *Response*: This sentence has been revised as suggested to enhance clarity and ensure the focus on communication skills development is evident.
  - Page 6, l 115: "intentionally crafted by our …" Suggestion: "intentionally developed …"
  - *Response*: We agree with this suggestion and have replaced the word "crafted" with "developed."
  - Page 6, line 118: "… and participating in online school." Suggestion: Clarify who was participating in the online school, as it could be confusing.
  - *Response*: We have clarified that it was the grandmother participating in an e-learning program, per the suggestion.

**Methods**:

- *Comment*: Did you pilot test the cases prior to the study? If not, please state. Also, were patients involved in the development?
  - *Response*: We pilot-tested the cases during the training session with the standardized patients, who provided feedback. However, the cases were not pilot-tested with the clinicians themselves. Additionally, the cases were developed by our team without direct input from patients. We believe we had not made this clear enough initially, and we have added these clarifications to our manuscript.
- *Comment*: How many SPs did you plan on training and to be part of the study? Please describe how you selected these and which criteria were applied.
  - *Response*: We have added the following additional language to the manuscript: "We trained approximately 8 standardized patients in a recorded session. These SPs were selected by the contracted standardized patient company primarily based on their availability and prior experience in telehealth simulation. Any SP who was not present for the in-person training session was able to view the session recording asynchronously to ensure consistency in their portrayal and in their grading of participants based on the checklist."
- *Comment*: Instrument Design: How did you develop the SP survey? Can you please provide us with a bit more information here, e.g., how many items, what is the foundation of the development? Also, did you test the instruments before using them in this study?
  - *Response*: The survey checklist was aligned with the learning objectives and modified from the Kalamazoo Essential Elements Communications Checklist. It consisted of 22 assessment items, focusing on communication skills that are essential for equitable telehealth care. The checklist was iteratively refined by the study team and pilot-tested internally with the SPs to assess feasibility and clarity. Answers to these questions have been included in the manuscript.
- *Comment*: Curriculum development and teaching: I am missing the section of how the curriculum looked and how it was taught. Please provide an additional section on this in your methods section.
  - *Response*: We have clarified that this was not a full curriculum but a standalone training session designed to be time-efficient for practicing clinicians. We focused on developing and testing whether such an intervention could be effective without extensive pre-session didactics, which we have noted in the updated manuscript.

**Results**:

- *Comment*: The way you present the results in Table 2 with the SE values as well as p-values is a bit unusual and confusing. Could you please present the results in a more standardized statistical way, e.g., not repeating SE within the cells/results, adding an additional p-value column, or marking significance by * or **?
  - *Response*: We have revised Table 2 to improve clarity. The updated table now has a dedicated p-value column, and significance is marked by an asterisk with annotations below the table. We believe this revision makes the data more accessible and standardized, and we thank the reviewer for the recommendations for how to format the data.
- *Comment*: I also have trouble understanding what "Percentage who performed" represents. Please clarify.
  - *Response*: We have revised the column title to "Percentage of Learners Who Completed Skill" to more accurately convey that it represents the percentage of learners who successfully performed the skill on that item of the checklist.
- *Comment*: I miss the t-value and degrees of freedom for the t-test. Could you please report these?
  - *Response*: We have included the t-values and degrees of freedom for all relevant comparisons in Table 2, as requested.
- *Comment*: I have the same feedback for Table 3. Please adjust to make the presentation clearer.
  - *Response*: We have revised Table 3 to present the data in a standardized way, similar to Table 2, to ensure clarity and readability.

**Discussion**:

- *Comment*: Will give peer review once the method and results sections are clear.
  - *Response*: We have made substantial revisions to the Methods and Results sections to improve clarity. We look forward to receiving additional feedback on the Discussion section.

**Reviewer #1 Additional Questions and Responses**

1. **Is the manuscript technically sound, and do the data support the conclusions?** *Reviewer #1*: Partly

*Response*: We have made significant revisions to the manuscript to ensure that the data presented clearly supports our conclusions. Specifically, we have refined our data presentation to align results with our research objectives and to provide a distinct separation between results and discussion. Additionally, we have included further details regarding our study methodology and data interpretation, as recommended.

2. **Has the statistical analysis been performed appropriately and rigorously?** *Reviewer #1*: I Don't Know

*Response*: We have updated our statistical analysis section to ensure that all relevant statistical tests are explicitly described. This includes providing t-values, degrees of freedom, and p-values for all analyses. We believe these changes will help clarify the rigor of our statistical approach.

3. **Have the authors made all data underlying the findings in their manuscript fully available?** *Reviewer #1*: No

*Response*: We included the Stata .dta file in the initial submission to ensure that all data underlying the findings are fully available. If the editorial team prefers a different manner of data availability, we are happy to comply to meet PLOS ONE's data policy and ensure transparency.

4. **Is the manuscript presented in an intelligible fashion and written in standard English?** *Reviewer #1*: Yes

*Response*: Thank you for this assessment. We have made additional language refinements based on Reviewer #1's suggestions to further improve clarity and readability.

We are grateful for the detailed feedback provided by the editorial team and Reviewer #1, and we believe that the revisions have considerably strengthened the manuscript.

**Additional Editorial Requirements**

**1. Journal Style Requirements**
*Editor Comment*: Please ensure that your manuscript meets PLOS ONE's style requirements, including those for file naming.

*Response*: We have reviewed our manuscript and made adjustments to ensure compliance with PLOS ONE's formatting and style requirements. The file names have also been updated to meet the journal's specifications.

**2. Funding Statement**
*Editor Comment*: Please provide an amended funding statement that declares all the funding or sources of support received during this study, whether external or internal, and include the statement: "There was no additional external funding received for this study."

*Response*: In the online interface, I am unable to make the requested adjustments to the financial disclosure statement. If the editor is willing to make this change on the back end for us, we would appreciate it. The amended funding statement should be as follows:

“This research was financially supported by the Association of American Medical Colleges Telehealth Equity Catalyst Grant (https://www.aamc.org/news/telehealth equity-catalyst-awards), grant number N/A, with authors CJN SEF and EMH as recipients. The funder had no role in study design, data collection and analysis, decision to publish, or preparation of the manuscript. There was no internal funding received for this study. There was no additional external funding received for this study.”

Thank you again for your time and consideration.

Sincerely,
Christopher J. Nash, MD, EdM
On behalf of all authors
